# Impact of electronic bedside meal ordering systems on dietary intake, patient satisfaction, plate waste and costs: A systematic literature review

**DOI:** 10.1111/1747-0080.12600

**Published:** 2020-01-19

**Authors:** Kristen MacKenzie‐Shalders, Kirsty Maunder, Daniel So, Rebecca Norris, Sally McCray

**Affiliations:** ^1^ Bond University Nutrition and Dietetics Research Group Faculty of Health Sciences and Medicine, Bond University Queensland Australia; ^2^ The CBORD Group Incorporated Sydney New South Wales Australia; ^3^ Faculty of Science, Medicine and Health University of Wollongong Wollongong New South Wales Australia; ^4^ Faculty of Medicine Nursing and Health Sciences Monash University Melbourne Victoria Australia; ^5^ Faculty of Medicine Nursingand Health Sciences Monash University, Central Clinical School, Department of Gastroenterology Melbourne Australia; ^6^ Dietetics and Foodservices Mater Health Brisbane Queensland Australia

**Keywords:** dietary intake, foodservice, patient satisfaction, systematic review, technology

## Abstract

**Aim:**

Hospital foodservices provide an important opportunity to deliver valuable dietary support to patients, address hospital‐acquired malnutrition risk and enhance patient satisfaction. Modifying the meal ordering process through the adoption of technology may actively engage patients in the process and provide an opportunity to influence patient and organisational outcomes. This systematic review was undertaken to evaluate the impact of electronic bedside meal ordering systems in hospitals on patient dietary intake, patient satisfaction, plate waste and costs.

**Methods:**

A systematic search following PRISMA guidelines was conducted across MEDLINE, CINAHL, EMBASE and Web of Science for randomised controlled trials and observational studies comparing the effect of electronic bedside meal ordering systems with traditional menus on dietary intake, patient satisfaction, plate waste and cost. The quality of included studies was assessed using the Quality Criteria Checklist for Primary Research tool.

**Results:**

Five studies involving 720 patients were included. Given the heterogeneity of the included studies, the results were narratively synthesised. Electronic bedside meal ordering systems positively impacted patient dietary intake, patient satisfaction, plate waste and costs compared with traditional menus.

**Conclusions:**

Despite the increase in healthcare foodservices adopting digital health solutions, there is limited research specifically measuring the impact of electronic bedside meal ordering systems on patient and organisational outcomes. This study highlights potential benefits of electronic bedside meal ordering systems for hospitals using traditional paper menu systems, while also identifying the need for continued research to generate evidence to understand the impact of this change and inform future successful innovations.

## 1 INTRODUCTION

There is an increasing focus within the hospital environment to provide quality care that enhances patient satisfaction and supports positive patient outcomes.^1^, ^2^ In the current consumer‐focused environment, hospital services aim to meet increasing patient expectations while simultaneously managing budgetary constraints and/or increasing expenses.^3^, ^4^ With a duty of care to provide safe, effective and equitable care to patients, hospitals must achieve this while treating and preventing malnutrition.^5^ Hospital foodservices provide a unique opportunity to influence dietary intake, address malnutrition risk and subsequent clinical outcomes across the hospital population. In addition, hospital foodservices are a key point of customer service and have the capacity to influence patients' perception of their entire hospital experience and enhance their satisfaction.^3^, ^6^, ^7^ Innovative foodservice models that enhance patient experience and improve dietary intake while reducing waste and remaining cost‐effective are therefore worthy of further investigation.

A potential tool to address these drivers is the utilisation of technology.^8^ While the adoption of technology in healthcare has been slower than other industries, electronic foodservice management systems have been increasingly implemented over the last decade to support food procurement, food preparation, meal ordering and delivery, allergen management and to enable foodservice model transformations, delivering positive patient and organisational outcomes.^3^, ^9^, ^10^ Customer‐focused technological innovations that can impact dietary intake and address malnutrition risk through enabling patients to be active participants in their meal ordering while in hospital, is the focus of this systematic review. Electronic bedside meal ordering systems (eBMOS) are used by meal ordering staff at the patient bedside on wireless devices, or by patients using bedside televisions/computers or their own mobile phone, to place their meal orders.^9^, ^10^ Any main meals or mid‐meals which the facility allows patients to have an advanced choice can be ordered via the eBMOS. This model is different to a traditional paper menu method of meal ordering (TM), as it enables real‐time patient data, including diet and allergies, to be available at the time of ordering. It also allows closer to mealtime ordering due to the data being entered directly into an electronic system ready for meal tray preparation.

To date, no systematic reviews have specifically evaluated the impact of eBMOS on patient and hospital outcomes in comparison to TM. It is important to understand whether this innovation is successfully delivering the outcomes it was designed to achieve, independent to the food delivery model, to guide hospitals in determining the best method for patient meal‐ordering. A recently published review assessing the impact of eBMOS had a broader inclusion criteria for the study design, did not require studies to include a comparator to the intervention and featured studies with concurrent changes in the foodservice system, such as a transformation to room service.^11^ Room service is well recognised as a foodservice model that can deliver improvements in hospital and patient outcomes, and therefore any improvements cannot be directly attributed to the utilisation of eBMOS. A high‐quality review published 5 years ago by Ottrey and Porter^3^ was also broader in scope than the current review and explored the effect of different menus and meal ordering systems on outcomes including dietary intake, cost, satisfaction and meal tray accuracy.

The aim of this systematic review was to (a) evaluate current empirical evidence on the impact of an eBMOS on key outcomes including patient dietary intake, patient satisfaction, plate waste and cost in comparison to a TM; and (b) review the quality of these studies using a validated tool. It is anticipated that this systematic review will provide an evidence‐base to uniquely inform future foodservice design relating to patient meal ordering models to positively benefit patient and organisational outcomes, as well as drive future research.

## 2 METHODS

This systematic literature review was undertaken in line with recommendations of the *Cochrane Handbook for Systematic Reviews of Interventions* 12 and reported according to the Preferred Reporting Items for Systematic Reviews and Meta‐Analysis: The PRISMA statement.^13^ The methodology for this review, including pre‐specified eligibility criteria and search strategies, was prospectively registered with the International Prospective Register of Systematic Reviews (CRD 42017059111).

A literature search was conducted in the online bibliographic databases MEDLINE (Ovid interface), CINAHL (EBSCO host interface), EMBASE (Elsevier interface) and Web of Science (Web of Knowledge portal) from inception to December 2018, with no date or language restrictions. Combinations of the terms “bedside menu ordering system,” “menu” and “hospital food service” were searched as medical subject headings and key or free text words. The search strategy is presented as **Appendix** **S1**. Additional relevant studies were retrieved through additional hand‐searching, contacting field experts and searching of ClinicalTrials.gov—a central repository of clinical trials—to identify ongoing studies.

Two authors for the first literature search (R.N., K.M.‐S.) and two for the updated search (D.S., K.M.‐S.) screened articles in a blinded, standardised manner with disagreements in judgement resolved by consensus or a third reviewer. Search results were exported to Endnote (X8; Thompson Reuters) and de‐duplicated prior to screening using the online screening application Rayyan.^14^ Following screening, full‐text manuscripts of potentially relevant studies were sought and reviewed. Studies were included if the following criteria was met: (a) prospective or retrospective observational study design, randomised controlled trial (RCT); (b) included adult participants (≥18 years of age); (c) took place in an acute healthcare/hospital setting; and (d) compared a new eBMOS with an existing TM. The term “eBMOS” was used by this review to describe an electronic solution for collecting patient meal orders.

Abstracts and non‐peer‐reviewed manuscripts were excluded. Studies that implemented and evaluated the use of room service or other broader foodservice model interventions were excluded.^15^, ^16^ Interventions that included a simultaneous change in foodservice models were excluded from the analysis as the outcomes could not be attributed to the meal ordering system alone.^15^, ^16^, ^17^, ^18^, ^19^

Review outcomes included the difference or change from the application of an eBMOS when compared to a comparator/control on the following outcomes: (a) patient dietary intake (defined as the amount of energy [kJ] and protein [g] consumed in a 24‐hour period and/or 48‐hour period); (b) plate waste (percentage of served food that remains uneaten by the patient^20^; (c) patient satisfaction (a subjective rating of hospital foodservices quality)^21^; or (d) cost (any cost associated with the food served, staff or overall system). A meta‐analysis was not considered appropriate due to the small number of eligible studies, which measured different outcomes using a range of tools.

The quality of included studies was evaluated by two independent reviewers (R.N. and D.S.) using the Quality Criteria Checklist for Primary Research tool from the Academy of Nutrition and Dietetics.^22^ To ascertain the presence or absence of threats to the validity of research, the tool consists of 10 questions encompassing: clarity of the research question; subject selection; comparability of study groups; handling of withdrawals; blinding; descriptions of the intervention; validity of outcome measures; appropriateness of data synthesis; conclusion support; and likelihood of funding bias.^22^ Based on these domains, overall quality ratings of positive (most validity questions answered yes, including the first four), neutral (one or more of the first four validity questions assessed as “no,” but other criteria indicate strengths) or negative (six or more of the domains are assessed as “no”) would be generated.^22^

## 3 RESULTS

**Figure 1:**
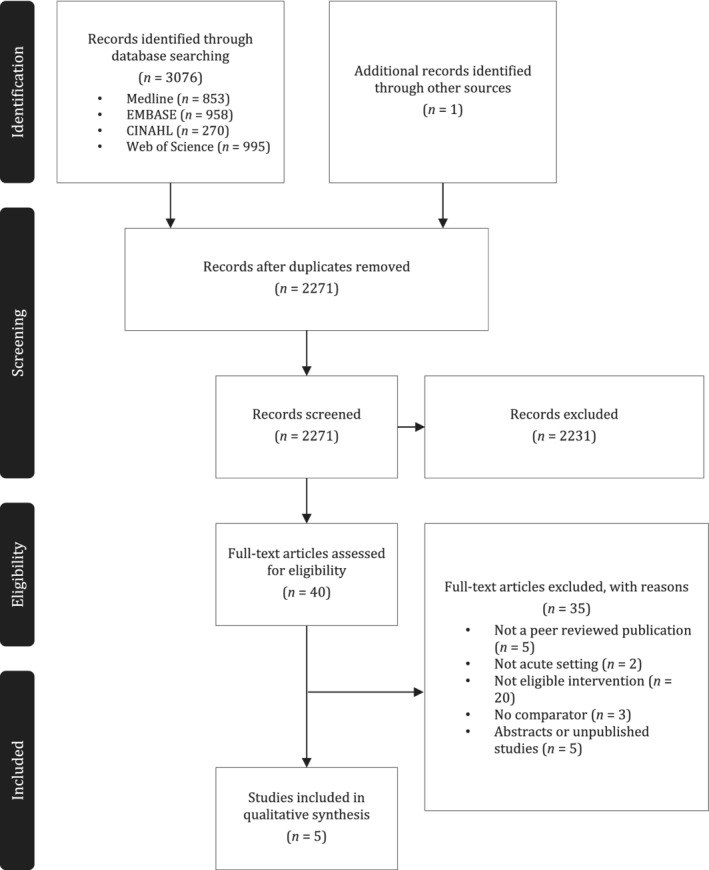
PRISMA diagram: Flowchart of studies included in the systematic review

A total of 3076 papers were retrieved from the database search for inclusion across the four online databases (Figure 1). Following the removal of duplicate papers (n = 805) and screening abstracts (n = 2270), 40 papers were retained for full text screening. One study was identified through hand‐searching, resulting in a total yield of five articles included in this review.

All studies compared an eBMOS to a TM (Table 1). Three studies evaluated the impact of a patient‐directed eBMOS (terminology including BMOS/e‐menu/TV menu)^10^, ^23^, ^24^ and the other two studies reported on staff‐deployed eBMOS.^9^, ^25^ One study was conducted using an observational point prevalence approach,^23^ with the remainder conducted using of pre‐ and post‐test study designs^9^, ^10^, ^24^, ^25^ (Table 1). Sample sizes investigated across included studies ranged from 50 participants to 860 participants.

**Table 1:** Characteristics table of studies evaluating the impact of electronic bedside menu ordering systems on foodservice and patient outcomes with a comparator

Author (year)	Country	Duration^a^	Cohort age^b^ (years)	n	Study design	Aim	Intervention; delivery	Comparator	Reported outcomes
Barrington et al (2018)	Australia	NA	Intervention: 65 Comparator: 61	201	Observational point prevalence	To determine changes in patient dietary intake, plate waste and meal experience associated with the implementation of a patient directed BMOS compared to traditional paper menus.	BMOS; Patient‐directed	Paper menu	Nutritional intake Plate waste Meal experience
Hartwell et al (2016)	UK	NA	68	162	Pre‐test post‐test	To evaluate an initiative in which e‐menus and touch screen technology were piloted in a large UK hospital.	E‐menu; Patient‐directed	Paper menu	Patient Satisfaction
Jamison et al (1996)	USA	NA	7‐78	50	Pre‐test, post‐test	To evaluate patient acceptability and cost‐effectiveness of a computerised menu selection system compared with that of a printed menu system.	Computerised menu (TV screen); staff‐deployed	Paper menu	Patient Satisfaction (acceptability) Cost effectiveness
Maunder et al (2015)	Australia	4	65	119	Quasi‐experimental pre‐test post‐test	To determine changes in the dietary intake and satisfaction of hospital patients, as well as the role of the NA, associated with the implementation of an electronic BMOS compared to a paper menu.	BMOS; staff‐deployed	Paper menu	Nutritional Intake Patient Satisfaction
McCray et al (2018)	Australia	NA	Intervention: 72 Comparator: 63	188	Observational point prevalence	To evaluate the impact of changing from a traditional paper menu ordering system to BMOS on key outcome measures of nutritional intake, plate waste, and the satisfaction of both patients and staff	BMOS; staff‐deployed	Paper menu	Nutritional intake Patient satisfaction Plate waste Food costs

Abbreviations: BMOS, Bedside Menu Ordering System; E‐menu, electronic menu; NA, not applicable; TV, television.

a Intervention duration in weeks; not applicable in study conducted using pre‐test, post‐test study designs.

b Age expressed in mean years of each group; age range provided when means were not obtainable; age expressed as entire cohort where per group data was not available.

The effect of eBMOS on dietary intake was reported in three studies. Barrington et al^23^ found that a patient‐directed eBMOS led to significantly higher mean daily energy intake 6457 ± 3069 kJ vs 4805 ± 2028 kJ (*P* < .001) and protein intake 72.3 ± 36.7 g vs 57.7 ± 26.9 g (*P* < .001) compared with a TM. Similarly, two staff‐deployed eBMOS models found a significantly higher mean daily energy intake compared with TMs 8273 ± 2043 kJ vs 6273 ± 1818 kJ (*P* < .001)^9^; and 6232 ± 2523 kJ vs 5513 ± 2212 kJ (*P* = .04).^25^ Likewise, these two studies also found mean daily protein intake was significantly higher with eBMOS compared with TMs 83 ± 24 g compared with 66 ± 25 g (*P* = .01)^9^; and 78 ± 36 g compared with 53 ± 24 g (*P* < .001).^25^ Further comparisons of energy and protein intake relative to the estimated requirements of patients were undertaken by Maunder et al^9^ and McCray et al.^25^ In the study undertaken by Maunder et al, patients receiving eBMOS met, on average, 110% estimated energy requirements and 105% estimated protein requirements compared with 86% for both using the traditional TM (*P* = .01 and *P* = .02, respectively).^9^ Similarly, McCray et al found that significantly more patients receiving eBMOS met their estimated energy (73% vs 64%; *P* = .02) and protein (98% vs 70%; *P* < .001) requirements compared with TM.^25^

Patient satisfaction for the overall hospital foodservice was assessed in three of the five papers^9^, ^10^, ^25^ (Table 2). Two studies showed that staff‐deployed eBMOS and TM reported high, stable scores in overall foodservice patient satisfaction using the Acute Care Hospital Foodservice Patient Satisfaction Questionnaire, which does not specifically explore satisfaction with the type of meal ordering system. Maunder et al^9^ reported patients rating their overall satisfaction as “good” or “very good” at 82% using eBMOS compared to 84% using the TM (*P* > .05). McCray et al^25^ also reported patients rating their overall satisfaction as “good” or “very good” at 74% using eBMOS and 75% with TM (*P* = 1.0). Hartwell et al^10^ evaluated satisfaction in a patient‐directed eBMOS compared to a TM across several domains (including temperature, presentation and ease of use), and reported the only difference was an increased satisfaction with regard to having meal ingredient information provided in eBMOS (*P* = .01).

**Table 2:** Summary of studies evaluating the effect of electronic bedside meal ordering systems on patient satisfaction

Author (year)	Intervention	Patient satisfaction tool	Tool validity	Satisfaction of intervention group (%)	Satisfaction of comparator group (%)	Overall satisfaction^a^
Barrington et al (2018)	Patient‐directed BMOS	KCFSQ	Y	46	54	NA^b^
Hartwell et al (2016)	E‐menu	10‐question survey	N	NA‐	NA	NA^b^
Jamison et al (1996)	Computerised menu	Two‐page survey	N	76	24	↑; P < 0.01
Maunder et al (2015)	BMOS^5^	ACHFPSQ; Meal Selection Survey	Y; N	82	84	→; P > 0.05
McCray et al (2018)	BMOS	ACHFPSQ; Meal Selection Survey	Y; N	65	35	→; P > 0.05

Abbreviations: ACHFPSQ, Acute Care Hospital Foodservice Patient Satisfaction Questionnaire; BMOS, Bedside Menu Ordering System; E‐menu, electronic menu; KCFSQ, King's College Food Service Questionnaire; NA, not applicable; N, no; Y, yes.

a Reported between group differences in patient satisfaction with overall hospital foodservice system.

b Between group differences in patient satisfaction not assessed.

Three studies assessed or asked specific additional questions related to patient satisfaction in regards to the new meal ordering system. Jamison et al^24^ found that patients preferred the eBMOS over the TM on the basis of interest, curiosity, convenience, availability, satisfaction and motivation (*P* < .01). When McCray et al^25^ and Maunder et al^9^ surveyed patients specifically about their menu ordering system preference, they found that significantly more preferred eBMOS to the TM in both studies; 84% vs 16% (*P* < .001)^25^ and 80% vs 15% with 6% not minding either way (*P* < .05).^9^ Two studies evaluated the effect of eBMOS on plate waste.^23^, ^25^ A patient‐directed model^23^ found no significant difference in average daily plate waste between BMOS (34.3%) and TM (35.4%) (*P* = .75), while a staff‐deployed model displayed a significant reduction in plate waste using eBMOS (30%) compared with TM (26%) (*P* < .001).^25^

Costs were evaluated in two studies.^24^, ^25^ McCray et al reported a decrease in total patient food cost of 19% for eBMOS compared with TM across a comparable 12‐month period.^25^ Jamison et al reported on the cost of effectiveness of implementation of the eBMOS determined by means of the payback method (ie, the time required to recoup the initial investment of their project). Costs were based on labour, software and printed menu costs for each model. They reported that operating the eBMOS instead of the TM would result in monthly savings of $1197 ($615 per month compared with $2093 per month) and an estimated payback period of 8.4 months.^24^ They also suggested additional possible savings could be achieved through a reduction in food waste due to increased accuracy of forecasting and tallying using the eBMOS.

**Figure 2:**
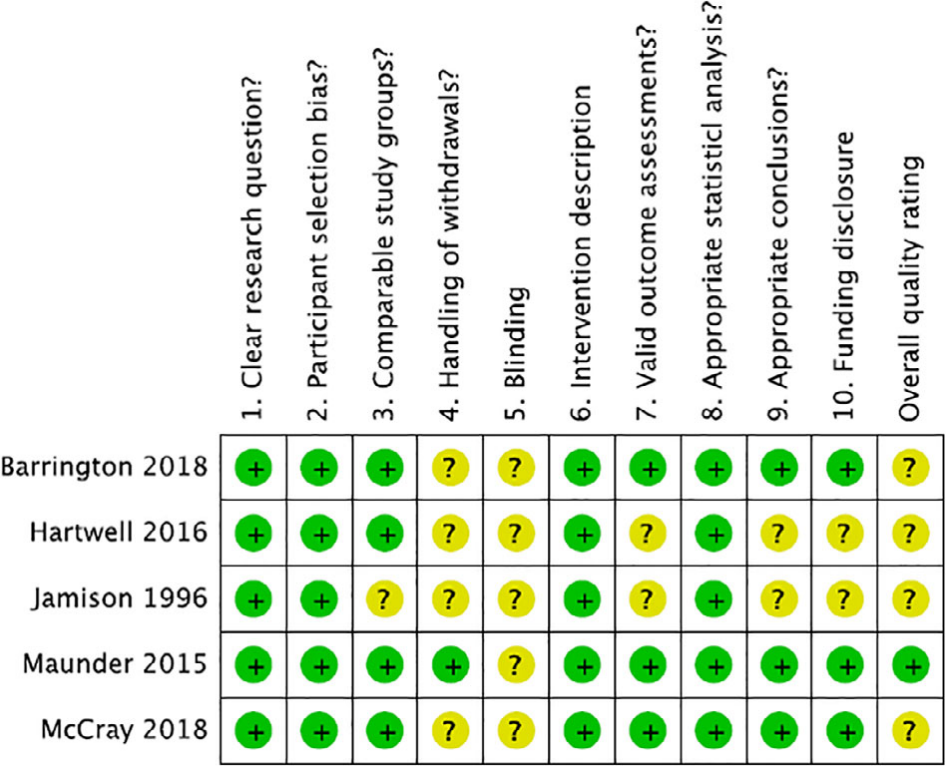
Quality Criteria Checklist and overall rating for each study included in this systematic review (n = 5). Risk of bias judgments performed per Primary Research Quality Criteria Checklist for Primary Research tool from the Academy of Nutrition and Dietetics.^22^ Plus/positive ratings presented as green/low; neutral ratings presented as yellow/unclear, minus/negative ratings presented as red/high

The overall quality of included studies was mostly neutral across the five included studies (Figure 2). The research question was clearly stated by all included studies, as were intervention descriptions, relevancy of study outcomes, specificity of inclusion criteria and analyses performed. The characteristics and subsequent comparability of stratified participant groups was adequately described in four studies,^9^, ^10^, ^23^, ^25^ while only one study discussed and response rates among participant groups.^9^ Three of the five included studies used validated methods to assess study outcomes.^9^, ^23^, ^25^ Although the conclusions of each study were supported by their results, limitations of the research were not considered in two studies.^10^, ^24^ Blinding for outcome assessments was not discussed in any of the included studies. Based on this risk of bias tool, the overall quality rating of included studies was mostly neutral: only a single study was judged as “positive”^9^ with the remainder assessed as “neutral.”^10^, ^23^, ^24^, ^25^

## 4 DISCUSSION

Despite the paucity of literature, this systematic literature review identified studies to demonstrate that an eBMOS has the potential to improve patient dietary intake and satisfaction, as well as reduce plate waste and foodservice costs. As healthcare continues to transition to a digital health environment, technological solutions that support consumer engagement, as well as provide essential patient and organisational benefits, will become critical in the future.

Three studies featured within the systematic review demonstrated that changing to an eBMOS can increase patients' dietary intake,^9^, ^23^, ^25^ which may consequently contribute to addressing malnutrition risk and preventing hospital‐acquired malnutrition.^26^, ^27^ This study refines the broader findings of complementary systematic reviews.^3^, ^7^, ^11^ While very specific in scope, it enables the opportunity to narrow the impact of other interventions and support the role of implementing an eBMOS as a core component of contributing to these positive outcomes. In each of these studies there was a major change in patient meal order timing, shifting from up to 24 hours in advance to between 1 and 4 hours prior to meals. Therefore, a potential explanation is that using an eBMOS facilitates patients to make meal orders closer to the mealtime, when they are more likely to know what they feel like eating, resulting in increased dietary intake. eBMOS may also enable more patients to receive their personal selections compared to TM, which is harder to manage patient dietary and location changes during their admission, and therefore may result in receiving standard default meals. While the calculations adopted for estimating dietary requirements were different across two studies and could have contributed to the differences in proportion of percentage of energy and protein requirements achieved,^9^, ^25^ there are other variables that can cause differences across sites, including the menu. However, the studies used consistent measures in the pre‐ and post‐data analysis within each study and found a consequent statistically significant increase in both studies of patients meeting their estimated energy and protein requirements when using eBMOS.

Patient satisfaction has long been a focus of achieving optimal foodservice models in healthcare, and systems and processes that encourage increased patient interaction and involvement with the meal order process have been suggested to improve satisfaction. This review featured several studies, albeit with small sample sizes, that showed that patient satisfaction was either maintained or improved after the implementation of eBMOS. To inform current and future meal ordering system design and to provide opportunities for research meta‐analysis, it may be useful to ensure consistency in use of a valid and reliable tool for measuring patient satisfaction with foodservices and specifically measuring satisfaction with the meal ordering process. Validated tools that measure patient satisfaction, for example, the Acute Care Hospital Foodservice Patient Satisfaction Questionnaire by Capra et al,^21^ are useful to assess overall satisfaction and are often related to food quality and potentially dietary intake but do not contain specific questions related to the meal ordering system or process. When surveys were conducted specifically around the meal ordering process, two studies found that the eBMOS was preferred over TM.^9^, ^25^

This ability of eBMOS to support closer to mealtime ordering may also have other positive effects; for example, it can decrease plate waste as evidenced in two studies within this review.^23^, ^25^ Other points of waste seen within a foodservice model such as duplicate trays produced for late meal orders due to poor and delayed communication of orders with a TM may be reduced using an eBMOS, as it enables real‐time information on patient status and meal orders. Oyarzun et al cited ineffective diet‐order communication as a major reason for late trays and accounting for 78% of extra meal trays required to be produced.^19^

While it is accepted that costs are a critical control for hospital foodservices, in this review only two studies reported a cost figure associated with changing their meal ordering system.^24^, ^25^ Additionally, one of these reviews was undertaken in 1996, before significant technological advancements.^24^ These two studies reported on different cost factors, one in relation to total patient food costs and the other on labour costs and time to take meal orders. Low costs reporting may be in part related to the fact that this information is sensitive or can be hard to measure and attribute impact to individual interventions. Nonetheless further information and clarity around cost measures will assist foodservice directors and managers to make informed decisions within budgetary constraints and be able to clearly demonstrate the financial impact of system and process changes.^28^ Interventions that utilise technology to provide improved communication regarding the meal order may assist in reducing overall waste and therefore costs.

The main strengths of this systematic review were its strict inclusion criteria ensuring that the intervention was predominantly related to a change to an eBMOS, and that studies with concurrent changes in their distribution system or other major foodservice systems were excluded. There are several limitations which should be considered when interpreting the findings of this review. A paucity of high‐quality studies of robust design that specifically answered the research question were identified and therefore a narrative synthesis of key findings was undertaken. Of the five studies that were included, one study received a positive score^9^ while four were assessed as neutral^10^, ^23^, ^24^, ^25^ using the Quality Criteria Checklist.^22^ A recent systematic review of foodservice interventions found that only nine of 33 included studies had sufficient methodological quality to meet evidence‐based scientific standards.^7^ Conducting foodservice research in an active hospital setting is challenging; however, investment in high‐quality, published foodservice research is essential to demonstrate the potential impact of foodservice innovations in influencing patient and organisational outcomes.^7^, ^9^, ^26^

This review provides the many hospitals utilising a TM evidence that transitioning to an eBMOS have the potential to improve dietary intake, patient satisfaction, plate waste and foodservice costs. There are now a range of cost‐effective technologies available to facilitate this process. As hospitals increasingly investigate technological opportunities to enhance their operation, communicating with facilities that have previously made similar changes, and piloting solutions can help to inform the feasibility, and manage risk.^7^ In addition, encouraging a research culture within foodservice dietetics, implementing system changes and innovations within a research framework, and collecting pre‐ and post‐implementation data using validated tools will continue to generate valuable evidence to inform future foodservice system interventions.

## CONFLICT OF INTEREST

K.M.‐S.: None to declare, contribution undertaken as part of employment at Bond University. K.M.: K.M. acknowledges the non‐financial support of her employer The CBORD Group. D.S.: None to declare, manuscript contribution as part of employment at Bond University. R.N.: None to declare, manuscript contribution as part of study at Bond University. S.M.: None to declare, manuscript contribution as part of employment at Mater Hospital.

## AUTHOR CONTRIBUTIONS

K.M.‐S.: Study design and concept, study protocol, second reviewer for initial and updated search, manuscript completion and submission. K.M.: Study design and concept, critical analysis and revision of manuscript. D.S.: Systematic literature search and screening, data extraction, risk of bias, revision of methodology. R.N.: Study protocol, systematic literature search and screening, data extraction, risk of bias, draft of manuscript. S.M.: Study design and concept, study protocol, critical analysis and revision of manuscript. All authors approved final version. The authors acknowledge David Honeyman, Bond University Librarian, for his contribution to the protocol and search strategy. All authors agree with the manuscript and declare that the content has not been published elsewhere.

The lead author affirms that this manuscript is an honest, accurate and transparent account of the study being reported. The reporting of this work is compliant with PRISMA guidelines. The lead author affirms that no important aspects of the study have been omitted and that any discrepancies from the study as planned have been explained.

## Supporting information


**Appendix S1**. Search strategies

## References

[1] Aase S . Hospital foodservice and patient experience: What's new? J Am Diet Assoc. 2011;111(8):1118‐1123. 21802554 10.1016/j.jada.2011.06.017

[2] Fallon A , Gurr S , Hannan‐Jones M , Bauer JD . Use of the Acute Care Hospital Foodservice Patient Satisfaction Questionnaire to monitor trends in patient satisfaction with foodservice at an acute care private hospital. Nutr Diet. 2008;65(1):41‐46.

[3] Ottrey E , Porter J . Hospital menu interventions: a systematic review of research. Int J Health Care Qual Assur. 2016;29(1):62‐74. 26771061 10.1108/IJHCQA-04-2015-0051

[4] White M , Wilcox J , Watson R , Rogany A , Meehan L . Introduction of a patient‐centred snack delivery system in a children's hospital increases patient satisfaction and decreases foodservice costs. J Food. 2008;19(3):194‐199.

[5] Agarwal E , Ferguson M , Banks M , Bauer J , Capra S , Isenring E . Nutritional status and dietary intake of acute care patients: results from the nutrition care day survey 2010. Clin Nutr. 2012;31(1):41‐47. 21862187 10.1016/j.clnu.2011.08.002

[6] Allison SP . Hospital food as treatment. Clin Nutr. 2003;22(2):113‐114. 12706126 10.1054/clnu.2002.0641

[7] Dijxhoorn DN , Mortier MJMJ , Van Den Berg MGA , Wanten GJA . The currently available literature on inpatient foodservices: systematic review and critical appraisal. J Acad Nutr Diet. 2019;119(7):1118‐1141. 31031106 10.1016/j.jand.2019.01.018

[8] Maunder K , Williams P , Walton K , Ferguson M , Beck E , Probst Y . Introduction to nutrition informatics in Australia. Nutr Diet. 2014;71(4):289‐294.

[9] Maunder K , Lazarus C , Walton K , Williams P , Ferguson M , Beck E . Energy and protein intake increases with an electronic bedside spoken meal ordering system compared to a paper menu in hospital patients. Clin Nutr ESPEN. 2015;10(4):e134‐e139. 28531390 10.1016/j.clnesp.2015.05.004

[10] Hartwell H , Johns N , Edwards JSA . E‐menus‐managing choice options in hospital foodservice. Int J Hosp Manag. 2016;53:12‐16.

[11] Prgomet M , Li J , Li L , Georgiou A , Westbrook JI . The impact of electronic meal ordering systems on hospital and patient outcomes: a systematic review. Int J Med Inform. 2019;129:275‐284. 31445267 10.1016/j.ijmedinf.2019.06.023

[12] Higgins J , Green S . Cochrane Handbook for Systematic Reviews of Interventions. Hoboken, NJ: Wiley; 2011.

[13] Moher D , Shamseer L , Clarke M , et al. Preferred reporting items for systematic review and meta‐analysis protocols (PRISMA‐P) 2015 statement. Syst Rev. 2015;4(1):1. 25554246 10.1186/2046-4053-4-1 PMC4320440

[14] Ouzzani M , Hammady H , Fedorowicz Z , Elmagarmid A . Rayyan—a web and mobile app for systematic reviews. Syst Rev. 2016;5(1):210. 27919275 10.1186/s13643-016-0384-4 PMC5139140

[15] Ottrey E , Porter J . Exploring patients' experience of hospital meal‐ordering systems. Nurs Stand. 2017;31(50):41‐51. 10.7748/ns.2017.e10435 28792334

[16] Sathiaraj E , Priya K , Chakraborthy S , Rajagopal R . Patient‐centered foodservice model improves body weight, nutritional intake and patient satisfaction in patients undergoing cancer treatment. Nutr Cancer. 2019;71(3):418‐423. 30260687 10.1080/01635581.2018.1506490

[17] McCray S , Maunder K , Krikowa R , Mackenzie‐Shalders K . Room service improves nutritional intake and increases patient satisfaction while decreasing food waste and cost. J Acad Nutr Diet. 2018;118(2):284‐293. 28676228 10.1016/j.jand.2017.05.014

[18] McCray S , Maunder K , Barsha L , Mackenzie‐Shalders K . Room service in a public hospital improves nutritional intake and increases patient satisfaction while decreasing food waste and cost. J Hum Nutr Diet. 2018;31(6):734‐741. 29989236 10.1111/jhn.12580

[19] Oyarzun VE , Lafferty LJ , Gregoire MB , et al. Evaluation of efficiency and effectiveness measurements of a foodservice system that included a spoken menu. J Am Diet Assoc. 2000;100(4):460‐463. 10767905 10.1016/S0002-8223(00)00141-3

[20] Walton K . Improving opportunities for food service and dietetics practice in hospitals and residential aged care facilities. Nutr Diet. 2012;69(3):222‐225.

[21] Capra S , Wright O , Sardie M , Bauer J , Askew D . The Acute Care Hospital Foodservice Patient Satisfaction Questionnaire: the development of a valid and reliable tool to measure patient satisfaction with acute care hospital foodservices. Food Res Int. 2005;16(1–2):1‐14.

[22] Academy of Nutrition and Dietetics . Evidence Analysis Manual: Steps in the Academy Evidence Analysis Process. Chicago, IL: Academy of Nutrition and Dietetics; 2016.

[23] Barrington V , Maunder K , Kelaart A . Engaging the patient: improving dietary intake and meal experience through bedside terminal meal ordering for oncology patients. J Hum Nutr Diet. 2018;31(6):803‐809. 29963727 10.1111/jhn.12573

[24] Jamison J , Bednar C , Alford B , Hsueh A . A computerized interactive menu selector system for hospitals. J Am Diet Assoc. 1996;96(10):1046‐1047. 8841169 10.1016/S0002-8223(96)00277-5

[25] McCray S , Maunder K , Norris R , Moir J , MacKenzie‐Shalders K . Bedside menu ordering system increases energy and protein intake while decreasing plate waste and food costs in hospital patients. Clin Nutr ESPEN. 2018;26:66‐71. 29908685 10.1016/j.clnesp.2018.04.012

[26] Agarwal E , Ferguson M , Banks M , et al. Malnutrition and poor food intake are associated with prolonged hospital stay, frequent readmissions, and greater in‐hospital mortality: results from the nutrition care day survey 2010. Clin Nutr. 2013;32(5):737‐745. 23260602 10.1016/j.clnu.2012.11.021

[27] Barker LA , Gout BS , Crowe TC . Hospital malnutrition: prevalence, identification and impact on patients and the healthcare system. Int J Environ Res Public Health. 2011;8(2):514‐527. 21556200 10.3390/ijerph8020514 PMC3084475

[28] Rodgers S . Selecting a food service system: a review. Int J Contemp Hosp Manag. 2005;17(2):147‐156.

