# Supplementary material for: Impact of electronic bedside meal ordering systems on dietary intake, patient satisfaction, plate waste and costs: A systematic literature review
Source: Nutr Diet. 2020 Jan 19;77(1):103–11. doi: 10.1111/1747-0080.12600 (PMC7383857; doi:10.1111/1747-0080.12600)
Supplement: Supplementary file 1 — Appendix S1. Search strategies [file NDI-77-103-s001.docx]

**Appendix S1. Search strategies**

December 15^th^, 2018

MEDLINE via Ovid

(menu*.tw. OR eMenu*.tw. OR ((food OR meal*) and order*).tw. OR Meals/ OR catering service*.tw. OR hospital food service*.tw. OR meal ordering system*.tw.)

(BMOS.tw. OR bed?side.tw OR spoken.tw OR electronic.tw. OR informatics.tw. OR system.tw. OR wireless.tw. OR computer*.tw. OR monitor.tw. OR digital.tw. OR exp Food Service/ OR exp Hospitals/)

(acute.tw. OR hospital*.tw. OR hospital patient*.tw.)

Results: 853

**Excerpta Medica Database (EMBASE) via Elsevier**

(menu*:ti,ab OR eMenu*:ti,ab OR ((food OR meal*) AND order*):de OR “meal”/exp OR “catering service”/exp OR “hospital food service”/exp OR “meal ordering system”/exp)

(BMOS:ti,ab OR bedside:ti,ab OR “bed side”:ti,ab OR spoken:ti,ab OR electronic:ti,ab OR informatics:ti,ab OR system:ti,ab OR wireless:ti,ab OR computer*:ti,ab OR monitor:ti,ab OR digital:ti,ab)

(acute:ti,ab OR hospital*:ti,ab OR “hospital patient*”:ti,ab)

Results: 958

**Cumulative Index to Nursing and Allied Health Literature (CINAHL) via EBSCO host**

(menu* OR eMenu* OR ((food OR meal*) AND order*) OR (MH “Meals”) OR (MH “Menu Planning”) OR (MH “Food Service Department”) OR meal ordering system*

BMOS OR bedside OR “bed side” OR spoken OR electronic OR informatics OR system OR wireless OR computer* OR monitor OR digital

acute OR hospital* OR (MH “Inpatients”) OR “hospital inpatient*”

Results: 270

**Web of Science via Web of Knowledge**

TI=((menu* OR eMenu* OR ((food OR meal*) AND order*) OR meal* OR catering service* OR hospital food service* OR meal ordering system*)) OR AB=((menu* OR eMenu* OR ((food OR meal*) AND order*) OR meal* OR catering service* OR hospital food service* OR meal ordering system*))

TI=((BMOS OR bedside OR bed side OR spoken OR electronic OR informatics OR system OR wireless OR computer* OR monitor OR digital)) OR AB=((BMOS OR bedside OR bed side OR spoken OR electronic OR informatics OR system OR wireless OR computer* OR monitor OR digital))

TI=((acute OR hospital* OR hospital inpatient*)) OR AB=((acute OR hospital* OR hospital inpatient*))

Results: 995
